# Single-Cell Deconvolution of Head and Neck Squamous Cell Carcinoma

**DOI:** 10.3390/cancers13061230

**Published:** 2021-03-11

**Authors:** Zongtai Qi, Yating Liu, Michael Mints, Riley Mullins, Reilly Sample, Travis Law, Thomas Barrett, Angela L. Mazul, Ryan S. Jackson, Stephen Y. Kang, Patrik Pipkorn, Anuraag S. Parikh, Itay Tirosh, Joseph Dougherty, Sidharth V. Puram

**Affiliations:** 1 Department of Otolaryngology-Head and Neck Surgery, Washington University School of Medicine, St. Louis, MO 63110, USA; qizongtai@wustl.edu (Z.Q.); rileymullins@wustl.edu (R.M.); r.a.sample@wustl.edu (R.S.); tlaw@wustl.edu (T.L.); tfbarrett@wustl.edu (T.B.); amazul@wustl.edu (A.L.M.); jackson.ryan@wustl.edu (R.S.J.); ppipkorn@wustl.edu (P.P.); 2 Department of Genetics, Washington University School of Medicine, St. Louis, MO 63110, USA; yliu41@wustl.edu; 3 Department of Molecular Cell Biology, Weizmann Institute of Science, Rehovot 7610001, Israel; michael.mints@weizmann.ac.il (M.M.); itay.tirosh@weizmann.ac.il (I.T.); 4 Clinical Research Training Center, Washington University School of Medicine, St Louis, MO 63110, USA; 5 Division of Head and Neck Oncology, Department of Otolaryngology—Head and Neck Surgery, The James Cancer Hospital and Solove Research Institute, The Ohio State University, Columbus, OH 43210, USA; Stephen.Kang@osumc.edu (S.Y.K.); Anuraag_Parikh@meei.harvard.edu (A.S.P.); 6 Department of Psychiatry, Washington University School of Medicine, St. Louis, MO 63110, USA; 7 Department of Otolaryngology, Massachusetts Eye and Ear, Boston, MA 02114, USA; 8 Department of Otolaryngology, Harvard Medical School, Boston, MA 02114, USA

**Keywords:** head and neck squamous cell carcinoma, deconvolution, single-cell RNA sequencing, regulatory T-cells

## Abstract

**Simple Summary:**

Tumors are not composed of a uniform ball of cells, but rather, a complex set of diverse cells. Unfortunately, most transcriptomic techniques analyze the entire tumor (bulk), and thus represent an average profile of genes expressed across heterogeneous cells. To estimate tumor composition from bulk data, many algorithms have been developed—broadly termed deconvolution. However, with the advent of single-cell RNA sequencing (scRNA-seq), which provides gene expression data for individual cells, a few deconvolution algorithms are now more nuanced. We have used our scRNA-seq data from head and neck tumors along with two cutting-edge deconvolution algorithms to analyze bulk expression data from >500 tumors. With this approach, we find that higher proportions of a class of immune cells (tumor-infiltrating regulatory T-cells) are associated with improved survival in head and neck cancer. Our findings and data establish a generalizable approach that can be applied across oncology to study tumor composition.

**Abstract:**

Complexities in cell-type composition have rightfully led to skepticism and caution in the interpretation of bulk transcriptomic analyses. Recent studies have shown that deconvolution algorithms can be utilized to computationally estimate cell-type proportions from the gene expression data of bulk blood samples, but their performance when applied to tumor tissues, including those from head and neck, remains poorly characterized. Here, we use single-cell data (~6000 single cells) collected from 21 head and neck squamous cell carcinoma (HNSCC) samples to generate cell-type-specific gene expression signatures. We leverage bulk RNA-seq data from >500 HNSCC samples profiled by The Cancer Genome Atlas (TCGA), and using single-cell data as a reference, apply two newly developed deconvolution algorithms (CIBERSORTx and MuSiC) to the bulk transcriptome data to quantitatively estimate cell-type proportions for each tumor in TCGA. We show that these two algorithms produce similar estimates of constituent/major cell-type proportions and that a high T-cell fraction correlates with improved survival. By further characterizing T-cell subpopulations, we identify that regulatory T-cells (T_regs_) were the major contributor to this improved survival. Lastly, we assessed gene expression, specifically in the T_reg_ population, and found that TNFRSF4 (Tumor Necrosis Factor Receptor Superfamily Member 4) was differentially expressed in the core T_reg_ subpopulation. Moreover, higher TNFRSF4 expression was associated with greater survival, suggesting that TNFRSF4 could play a key role in mechanisms underlying the contribution of T_reg_ in HNSCC outcomes.

## 1. Introduction

Head and neck squamous cell carcinoma (HNSCC) arises in the upper aerodigestive mucosa of the oral cavity, oropharynx, hypopharynx, larynx, and rarely, in the nasal cavity and nasopharynx [1]. Together, HNSCC is the sixth most common cancer worldwide, representing 90% of cancers that arise in the head and neck region [2,3] and accounting for 650,000 (3.6%) of cancer cases and 330,000 (3.4%) of cancer deaths per year [2]. Unfortunately, the prognosis for HNSCC patients remains poor despite numerous advances in surgical, radiation, and systemic therapies [4]. Advancing new therapeutics for HNSCC will require insight into the cellular and molecular biology underlying HNSCC.

Knowledge of cell-type composition in tumor tissues represents an important step towards identifying cellular targets in cancer. Changes in cell composition underlie diverse physiological states of complex tissues. In malignant tumors, levels of infiltrating immune cells are associated with tumor growth, cancer progression, and patient outcomes [5,6]. However, beyond knowing the proportions, understanding how individual cell types respond may also be important for understanding the course of disease. For example, genes changes within particular cells might provide insights into novel avenues for treatment [6,7,8,9].

Intra-tumoral cell proportions can be estimated by histological techniques [10]. However, such approaches are low throughput, time-consuming methods that are not feasible to use with large sample sizes due to the requirement for specific antibodies and the limited number of cell types that can be simultaneously assessed [11]. By contrast, genomic (whole exome and whole genome sequencing; WES and WGS) and transcriptomic (RNA-seq) sequencing are high-throughput methods for analyzing bulk tumor samples which are suited for large sample sizes, such as The Cancer Genome Atlas (TCGA) dataset [12]. Although bulk sequencing approaches have identified driver mutations and abnormal expression profiles characteristic of HNSCC [13], these methods fail to capture intra-tumoral heterogeneity [1,14,15], which affects clinical outcomes and treatment response in HNSCC and other solid cancers [16,17,18,19,20]. Thus, improved therapies for HNSCC critically depend on the elucidation of the key cellular subpopulations present within these heterogeneous tumors.

Computational methods for the quantitative phenotyping of tumors from bulk RNA-seq data have significant potential for efficient and low-cost profiling of a large number of existing samples yet are handicapped by the intrinsic limitations of the bulk data itself. Single-cell RNA-sequencing (scRNA-seq) technology provides a promising alternative, providing high-resolution gene expression data for individual cells within a tumor [9,21]. Indeed, scRNA-seq is increasingly being utilized across oncology, but this method is constrained by the need for freshly acquired patient samples, significant expense, and technical difficulties in tissue processing [14,15,22]. Fortunately, a relatively small subset of existing scRNA-seq data can provide detailed cell-type-specific gene expression profiles that can be inputted into deconvolution algorithms. These algorithms enable cell-type proportion estimates and even a deciphering of cell-type-specific gene expression from bulk sequencing data [8,11,12,14,15,23,24,25]. Thus, deconvolution algorithms based on scRNA-seq-derived gene expression profiles overcome the resource limitations of scRNA-seq by permitting in silico cell-type-specific analyses from bulk tissue. This approach can enable large-scale investigation of novel or poorly characterized cell states in bulk tissue profiles and enable us to test new hypotheses in the substantial existing bulk profile collections. In human cancers, cellular states of interest may include subpopulations of activated, resting, or exhausted T-cells [26,27,28], cancer-associated fibroblasts [8], or malignant cells [29,30], including tumor-initiating cells or cancer stem cells [31].

Here, we sought to test the hypothesis that the proportions of individual cell types influence disease progression and outcome. We leveraged bulk transcriptomic data from >500 HNSCC samples profiled by TCGA. We derived the signature matrix from our previously profiled transcriptomes of ~6000 single cells from 21 head and neck squamous cell carcinoma (HNSCC) samples, including 4 matched pairs of primary tumors and lymph node metastases [8]. With this scRNA-seq reference, we used two recently developed deconvolution algorithms—CIBERSORTx [32] and MuSiC [33]—which allow the use of scRNA-seq as reference to characterize cell-type compositions from bulk RNA-seq data in complex tissues, to estimate immune and non-immune cell-type proportions in HNSCC TCGA bulk RNA-seq data. By correlating proportions of each cell type with patient-matched overall survival, we identified that high proportions of T-cells are associated with improved overall survival from HNSCC. By further characterizing T-cell subpopulations, we found that regulatory T-cells (T_regs_) are the major contributor to this superior survival. Lastly, we assessed the specific gene expression in the T_reg_ subpopulation and found that TNFRSF4 (Tumor Necrosis Factor Receptor Superfamily Member 4) is differentially expressed in core T_regs_ and is correlated with significantly better survival, raising the possibility that this gene could play a key role in the mechanisms underlying T_regs_ in HNSCC.

## 2. Results

### 2.1. Overview of Deconvolution Approach

**Figure 1:**
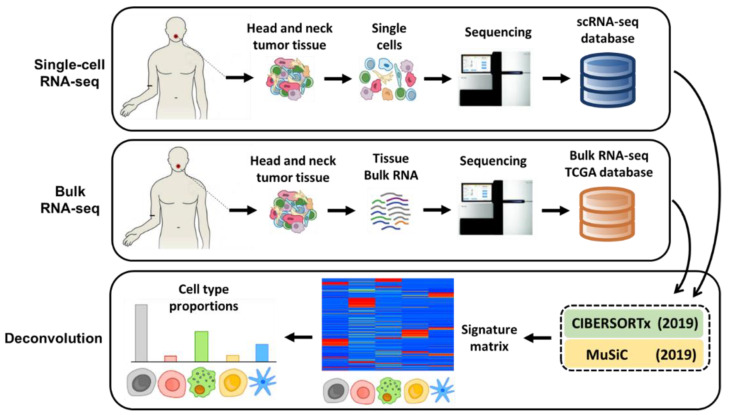
Schematic illustration of the bulk RNA-sequencing (RNA-seq) deconvolution with single-cell reference profile. A schematic overview of deconvolution analysis is illustrated. First, we derived a cell-type expression matrix from our previously profiled transcriptomes of ~6000 single cells by SMART-seq2 protocol from 21 HNSCC (Head and Neck Squamous Cell Carcinomas) samples, including four matched pairs of primary tumors and lymph node metastases. This established a benchmark for cell-type proportions in heterogenous HNSCC tissue. Second, we obtained the bulk RNA-seq data from ~500 HNSCC samples within TCGA. Lastly, we used either CIBERSORTx or MuSiC to derive a signature matrix and then deconvolve the bulk RNA-seq data to get cell proportions.

A schematic overview of our deconvolution approach is illustrated in Figure 1. First, we derived a cell-type expression matrix from our previously profiled transcriptomes of ~6000 single cells using the SMART-seq2 protocol [34] from 21 HNSCC samples [8]. This matrix established a benchmark for cell-type proportions in heterogeneous HNSCC tissue. Second, we obtained the bulk RNA-seq data from >500 HNSCC samples within TCGA and then used both CIBERSORTx [32] and MuSiC [33] to deconvolve the bulk RNA-seq data based on the derived cell-type expression matrix.

### 2.2. CIBERSORTx Analysis with scRNA-seq Reference for Nine Major Cell Types

**Figure 2:**
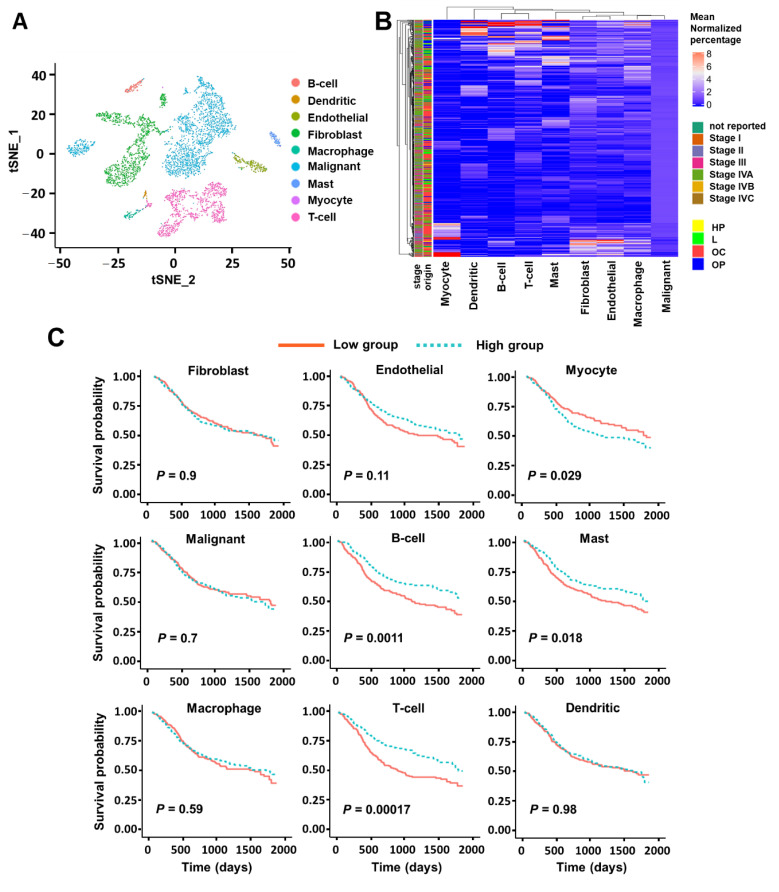
CIBERSORTx analysis with scRNA-seq reference of nine major cell types. (**A**) t-SNE projection of scRNA-seq data from 21 HNSCC samples colored by nine major cell clusters. (**B**) Heatmap of the relative cell fractions of the nine major cell types for each sample estimated by CIBERSORTx. The percentage is normalized by the corresponding mean within each cell type. The tissue origin and tumor stage are annotated as side bars. (HP = Hypopharynx; L = Larynx; OC = Oral Cavity; OP = Oropharynx). (**C**) Association between cell proportions and overall survival in patients with HNSCC profiled by TCGA. Estimated cell proportions were stratified by a half–half split, and the separation between survival curves was evaluated using a log-rank test.

CIBERSORTx is a computational framework to infer cell-type abundance and cell-type-specific gene expression from RNA profiles of intact tissues. CIBERSORTx had been validated in HNSCC using simulated tumors reconstructed from single cells [32]. To further utilize this algorithm to deconvolute bulk RNA-seq data in HNSCC, two inputs are required. One input is the gene expression dataset representing a bulk admixture of different cell types, which we obtained from TCGA [35]. The second input is the single-cell reference that enumerates the genes defining the expression profile for each cell type of interest, for which we used our previously profiled transcriptomes of ~6000 single cells [8]. To assign cell types to single cells, we generated a t-SNE (t-distributed Stochastic Neighbor Embedding) projection of scRNA-seq data revealing nine major cell clusters, which were further annotated by the expression of known marker genes for T-cells, B cells, macrophages, dendritic cells, mast cells, endothelial cells, fibroblasts, malignant cells, and myocytes (Figure 2A). Next, we used the cell-type versus gene matrix to generate the signature matrix by CIBERSORTx (Supplementary Materials Figure S1A). With this signature matrix, we estimated the cell fractions for each sample of bulk RNA-seq in TCGA using the CIBERSORTx algorithm, which revealed a wide range of cell-type proportions from ~80% malignant cells to ~2% T-cells (Supplementary File S1). The relative percentages of each cell type (normalized by the corresponding mean within each cell type) are shown via heatmap (Figure 2B).

To examine the association of tumor origin and stage with cell-type proportions, we performed hierarchical clustering of samples with nine type proportions and found no clear separation of origin or stage (Figure 2B). We then explored the association with individual cell proportions and found that oropharynx tumors were highly correlated with immune cells’ proportions, especially B cells and T cells (Supplementary Figures S2 and S3), which has been previously reported [12]. To further explore the association of tumor subtype with cell-type proportions, we first merged the subtype information [12] with our deconvolution result by TCGA ID and then we did the same hierarchical clustering analysis. We observed a strong association of the atypical subtype with three immune cell proportions: B cell, T cell, and Dendritic (Supplementary Figure S4). This association is expected because the vast majority of atypical subtype tumors are HPV (Human Papillomavirus) -positive oropharynx patients, which tend to have more immune infiltrate [36]. We also found that fibroblast and endothelial proportions were positively correlated with mesenchymal subtype and negatively correlated with basal subtype (Supplementary Figure S5), while malignant proportions were positively correlated with basal subtype and negatively correlated with mesenchymal subtype (Supplementary Figure S5), consistent with observations we made previously [8].

Next, we investigated the association of various cell-type proportions with survival in HNSCC patients. We began by classifying cell-type proportions into two groups, high and low, with an equal number of patients in each group. We calculated the survival for each group using the Kaplan–Meier log-rank test with corresponding Kaplan–Meier curves. A higher proportion of T-cells and B-cells was associated with more favorable survival with a *p*-value of less than 0.005 (Figure 2C). By contrast, there was no significant difference in survival among patients with high and low proportions of fibroblasts, endothelial cells, malignant cells, macrophages, or dendritic cells. Interestingly, a higher proportion of myocytes was associated with inferior survival with a *p*-value less than 0.05. This is consistent with a recent study [37] demonstrating that patients of tongue squamous cell carcinoma with high relative MEF2C (myocyte enhancer factor 2C) expression had an inferior overall survival. To address potential confounders of patient survival, a multivariate Cox proportional-hazards model was performed to identify independent prognostic factors for HNSCC survival. After adjusting for tumor stage, race, smoking status, and age, we confirmed that T-cell and B-cell proportions serve as independent prognostic parameters for overall survival (T-cell HR (Hazard Ratio): 0.63, *p* < 0.05; B-cell HR: 0.59, *p* < 0.05) in HNSCC patients, as shown in Table 1 and Table 2. To rule out that the effect of T cell proportion on survival is secondary to B cell, we correlated the estimated proportion of B-cell with that of T-cell (Supplementary Figure S6). The Pearson correlation coefficient is 0.42, indicating there is not a linear relationship between these two cell populations. Therefore, T cell and B cell are independent proxies.

### 2.3. CIBERSORTx Analysis with T-Cell Subtypes/Subpopulations

**Figure 3:**
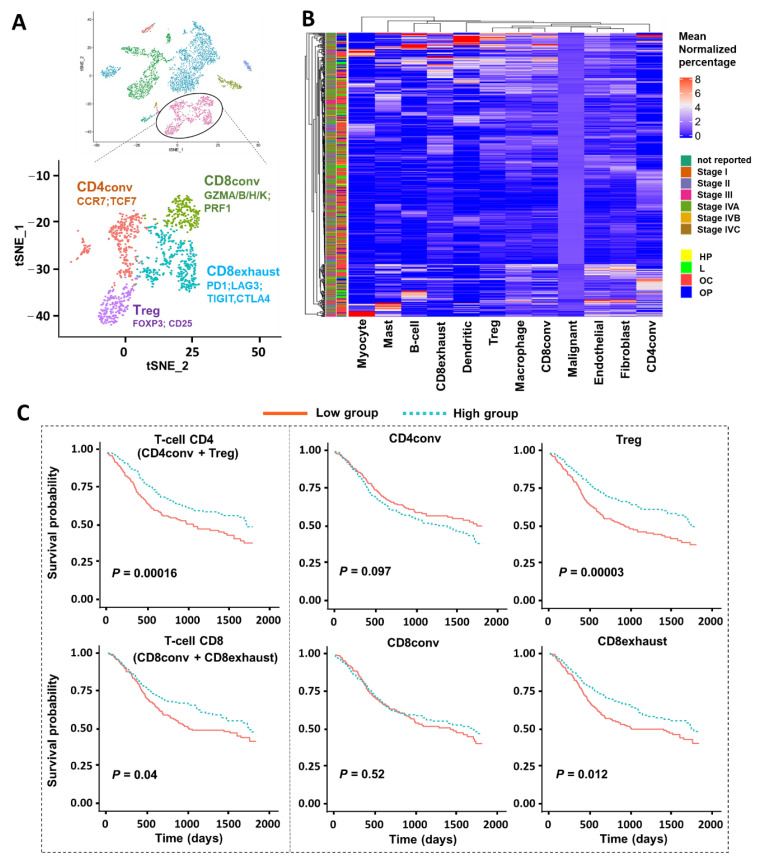
CIBERSORTx analysis based on T-cell subtypes/subpopulations. (**A**) t-SNE plots of T-cell population colored by four subtypes based on the corresponding marker genes: conventional CD4 T-cells (CD4conv; CCR7, TCF7), regulatory T-cells (T_reg_; FOXP3, CD25), conventional CD8 T-cells (CD8conv; GZMA/B/H/K, PRF1), and CD8 exhausted T-cells (CD8exhaust; PD1, LAG3, TIGIT, CTLA4). (**B**) Heatmap of the relative cell fractions of the 12 cell types (eight major cell types and four T-cell subtypes) for each sample estimated by CIBERSORTx. The percentage is normalized by the corresponding mean within each cell type. The tissue origin and tumor stage are annotated as side bars. (HP = Hypopharynx; L = Larynx; OC = Oral Cavity; OP = Oropharynx). (**C**) Association between cell proportions and overall survival in patients with HNSCC profiled by TCGA. Estimated cell proportions were stratified by a half–half split, and the separation between survival curves was evaluated using a log-rank test. The T-cell CD4 has two subtypes: conventional CD4 cells and regulatory T-cells (T_regs_). The T-cell CD8 population includes conventional CD8 T-cells and exhausted CD8 T-cells.

Given that a higher T-cell proportion was associated with improved survival in HNSCC patients and that our prior scRNA-seq dataset included a relatively large number of T-cells (~1000 T-cells), we next examined T-cell subtypes by finer clustering, producing four sub-clusters annotated by marker genes [8] as conventional CD4 T-cells (CD4_conv_; CCR7, TCF7), regulatory T-cells (T_regs_; FOXP3, CD25), conventional CD8 T-cells (CD8_conv_; GZMA/B/H/K, PRF1), and exhausted CD8 T-cells (CD8_exhausted_; PD1, LAG3, TIGIT, CTLA4) (Figure 3A). With the addition of T-cell subtypes, we created and derived a new signature matrix of 12 cell types (eight major cell types and four T-cell subtypes) by CIBERSORTx (Supplementary Figure S1B). We estimated the prevalence of these 12 cell types in the same TCGA bulk RNA-seq data as previously used (Figure 3B and Supplementary File S2) and then associated the proportions (low or high) of T-cell subtypes with patients’ survival outcomes by Kaplan–Meier curves and log-rank test (Figure 3C). We specifically examined the four T-cell subtypes: CD4_conv_ and T_reg_ under the umbrella of CD4 T-cells, and CD8_conv_ and CD8_exhausted_ under the category of CD8 T-cells. These results showed that higher proportions of CD4 T-cells were associated with favorable survival outcomes with a much lower *p*-value than that of CD8 T-cells (*p* = 0.00016 for CD4 T-cells, *p* = 0.04 for CD8 T-cells). Interestingly, T_regs_ demonstrated a significantly lower *p*-value (*p* = 0.00003) than that of the other three subtypes, suggesting that T_regs_ have a stronger association with improved survival in HNSCC than other T cell subsets. To rule out that T_regs_ serve as an indirect or secondary contributor since T_regs_ negatively regulate CD8 T cells, we noted that the *p*-value for CD8 T-cells’ effect on survival was not as significant as for T_regs_. In addition, if the effect on survival was primarily driven by T_regs_ negatively regulating CD8 T-cells, we might expect the CD8 and T_reg_ effects on survival to be opposed (i.e., high T_regs_ and low CD8 T-cells associated with improved survival). These observations directed our primary motivation for focusing on T_regs_. We next performed multivariate analyses with the Cox proportional-hazards model to identify the independent prognostic factors for HNSCC survival. After adjusting for tumor stage, patient race, smoking status, and age, we found that T_reg_ proportion is an independent predictor of overall survival (Table 3, HR: 0.61, *p* < 0.05).

### 2.4. MuSiC Deconvolution Based on T-Cell Subtypes/Subpopulations

**Figure 4:**
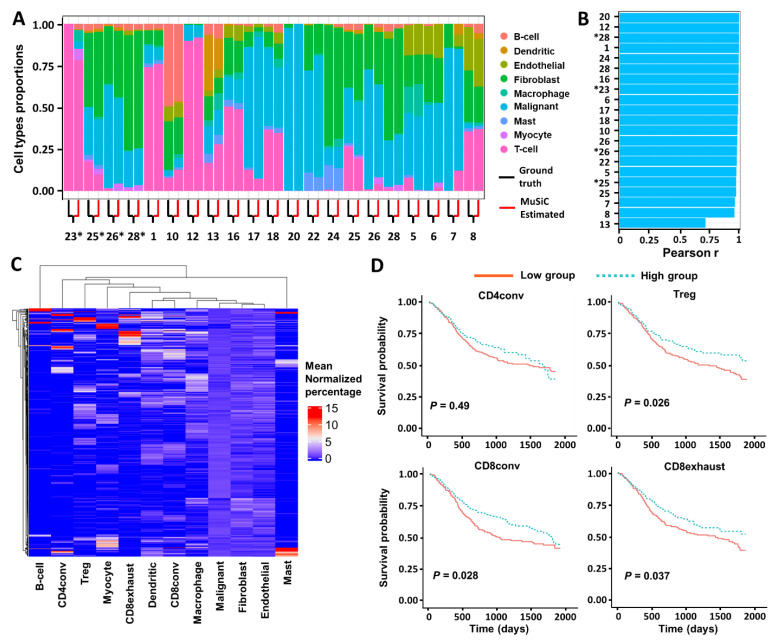
MuSiC deconvolution based on T-cell subtypes/subpopulations. (**A**) Comparison of ground-truth cell proportions with the estimated proportions by MuSiC for all HNSCC samples. The validation run used a combination of 18 (reference) and 3 (validation). The ground-truth and estimated cell proportions are paired for each sample and demarcated by black and red lines, respectively. Sample tumor numbers shown with an asterisk (*) indicate metastatic samples. (**B**) Concordance between cell type proportions measured by scRNA-seq (ground truth) and MuSiC for all HNSCC samples. The validation run used a combination of 18 (reference) and 3 (validation). The correlation is calculated by Pearson method and samples have the same naming criteria as in (**A**). (**C**) Heatmap of the relative cell fractions of the 12 cell types (eight major cell types and four T-cell subtypes) for each sample estimated by MuSiC. The percentage is normalized by the corresponding mean within each cell type. (**D**) Association between cell proportions and overall survival in patients with HNSCC profiled by TCGA. Estimated cell proportions were stratified by a half–half split, and the separation between survival curves was evaluated using a log-rank test.

To confirm that the T_reg_ subpopulation is associated with improved survival in an orthogonal approach, we used a separate but similar deconvolution algorithm known as multi-subject single-cell deconvolution (MuSiC) to validate our results. MuSiC incorporates cross-subject and cross-cell consistency of marker genes into the deconvolution algorithm, which allows for scRNA-seq datasets to serve as effective references for independent bulk RNA-seq datasets involving distinct patients. We first tested the deconvolution performance of MuSiC on simulated bulk-RNA-seq data reconstructed in silico from scRNA-seq that predetermines ground truth cell proportions. To have all 21 samples validated and avoid overlapping samples in each validation run, we utilized two combinations: 18 (reference) versus 3 (validation) and 14 (reference) versus 7 (validation). For example, as we have a total of 21 scRNA-seq samples, we used 18 of them to create the signature matrix and the remaining three to construct in silico bulk RNA-seq data. This approach yielded a total of seven (21/3 = 7) and three (21/7 = 3) validation runs for combinations of 14 versus 7 and 18 versus 3, respectively. We then ran each of the cases through MuSiC and compared the estimated cell proportions with the ground truth (Figure 4A and Supplementary Figure S7A). We found that the estimates aligned very well with the ground truth cell proportions: Patients were highly concordant with ground truth by Pearson correlation (Figure 4B and Supplementary Figure S7B). Strong performance was also maintained when considering deconvolution results across distinct cell types (except myocytes possibly due to limited scRNA-seq representation) (Supplementary Figure S7C,D).

After validation of the MuSiC method in our previous HNSCC dataset, we deconvolved the TCGA bulk RNA-seq data with nine major cell types (Supplementary Figure S8A and File S3). To evaluate how well the cell type compositions from these two algorithms align with each other, we calculated the Pearson correlation between the estimated cell proportions from CIBERSORTx with that of MuSiC. Patients were highly concordant between these two algorithms, with a median Pearson correlation coefficient of 0.96 (Supplementary Figure S9A). However, when checking the cell types, we found correlation coefficients are in discrepancies ranging from Myocyte 0.95 to Mast 0.18 (Supplementary Figure S9B). We focused on results most consistent across algorithms (i.e., correlation coefficient > 0.8). Therefore, four cell types (myocyte, T cell, Malignant, and Fibroblast) are cross-validated by these two algorithms.

Next, we further deconvolved the TCGA bulk RNA-seq data with 12 cell types (eight major cell types and four T-cell subtypes), as described above (Figure 4C and Supplementary File S4). Similar to the results obtained by CIBERSORTx, we found that a higher T-cell and B-cell proportion was associated with improved overall survival (Supplementary Figure S8B). The proportions of other cell types, including fibroblasts, macrophages, dendritic cells, endothelial cells, malignant cells, myocytes, and mast cells, were not associated with a significant difference in survival. Within the T-cell subtypes, we again observed that a higher proportion of T_regs_ was associated with improved survival (Figure 4D). We then performed multivariable analyses again and found similar results to CIBERSORTx, namely, that T_reg_ proportion is an independent predictor of overall survival (Table 3, HR: 0.70, *p* < 0.05).

### 2.5. CIBERSORTx Analysis of Gene Expression of Regulatory T-Cells

**Figure 5:**
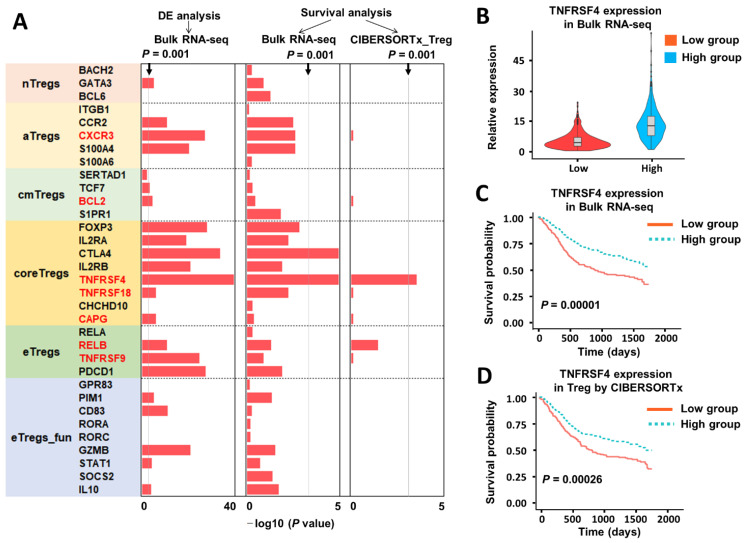
CIBERSORTx analysis of gene expression of regulatory T-cells (T_reg_). (**A**) Bar plot of the *p*-values calculated from differential gene analysis of low and high group defined by T_reg_ proportions, log-rank tests for survival outcomes of the low and high fraction groups of T_reg_ marker genes. In the first column, gene expression is obtained from bulk RNA-seq in TCGA. In the second column, gene expression is from the T_reg_ subpopulation estimated by CIBERSORTx. Genes marked in red have imputed values noted. Gray lines represent the *p*-value threshold (*p* = 0.001). (**B**) Violin plot of TNFRSF4 expression in low and high group defined by T_reg_ proportions. (**C**) Association between TNFRSF4 expression and overall survival in patients with HNSCC profiled by TCGA. Estimated cell proportions were stratified by a half–half split, and the separation between survival curves was evaluated using a log-rank test. (**D**) Association between T_reg_-specific TNFRSF4 expression estimated by CIBERSORTx and overall survival in patients with HNSCC profiled by TCGA. The same calculation method is performed as in (**C**).

To complement our cell-proportion-centric analyses, we conducted gene-centric differential expression analysis, survival analysis, and identified prognostic associations of marker genes that could potentially define specific subtypes and states of T_regs_ [38]. We first obtained the genome-wide expression values from bulk RNA-seq data as previously described and split them into halves by the T_reg_ proportion. We used the Wilcoxon signed rank test to identify differentially expressed genes and the adjusted *p*-values are plotted in Figure 5A for all 33 marker genes. We then split the genes into halves by expression values and used Kaplan–Meier curves to display survival distributions for each marker gene (Supplementary Figure S10 and Figure 5C). The log-rank test was used to assess the difference between patients with high and low values of the corresponding genes and the *p*-values are plotted in Figure 5A. Two genes, CTLA4 and TNFRSF4, passed the threshold (*p* < 0.001). We further examined the expression of all 33 marker genes, specifically among T_regs_ (rather than among all cells), using CIBERSORTx high-resolution mode. This analysis can impute genes’ expression to define distinct subpopulations, although marker genes with continuously high expression may not be imputed due to lack of statistical power. We were able to impute the expression for seven genes with this approach, and two genes, TNFSF4 and RELB, were differentially expressed in core and effector T_regs_, respectively (Supplementary File S5). We then associated the expression of these seven genes with overall survival (Figure 5A,D) and found that TNFRSF4 is the only one that passed the *p*-value threshold. We also show the differential expression of TNFRSF4 between the high and low group defined by T_reg_ proportions in Figure 5B. Combined with the results from bulk RNA-seq, TNFRSF4 may be a marker gene of particular interest. Next, we estimated the hazard ratios (HRs) for the risk of disease progression and mortality associated with high and low expression of TNFRSF4 in all cells and T_regs_ using the Cox proportional-hazards model. The results from all cells and T_regs_ agreed with each other and showed that high expression of TNFRSF4 is correlated with a significantly lower hazard of death (Table 4). Taken together, these data suggest that TNFRSF4 is differentially expressed in the core T_reg_ subset and is correlated with significantly better survival, indicating that this gene could play a key role in the mechanisms underlying the contribution of T_reg_ in HNSCC outcomes.

## 3. Discussion

In this study, we deconvoluted bulk RNA-seq data from >500 HNSCC samples profiled by TCGA using scRNA-seq data to define cell-type proportions and determine their association with survival. Heterogeneity among HNSCC patients appears highly relevant, with the proportions of several cell types strongly associated with survival. Specifically, a higher proportion of infiltrating T_regs_ is associated with improved outcomes, suggesting T_reg_ fraction may be an independent prognostic factor in HNSCC outcomes.

We have implemented two distinct but similar deconvolution algorithms, CIBERSORTx and MuSiC, to cross-validate the deconvolution results. Both methods allow the integration of sorted bulk data or scRNA-seq to derive a signature matrix. Compared with fluorescent-activated cell sorting (FACS)-purified or in vitro cell subsets, scRNA-seq does not rely on predetermined cell-type specific genes based on *a priori* knowledge, and therefore, enables unbiased transcriptional profiling of thousands of individual cells to guide cell-type-specific gene signatures. Moreover, it is now known that even ‘sorted or purified’ cells may still contain significant cellular heterogeneity [39]. The scRNA-seq-derived signature matrix thus captures a more comprehensive picture of cell diversity in heterogeneous HNSCC tissue. This approach represents a major advantage over past deconvolution techniques, which primarily rely on using a sorted bulk expression profile from one tissue type to analyze bulk data usually from an entirely different tissue type. By contrast, we used scRNA-seq from HNSCC patients to deconvolute the bulk RNA-seq data from an orthogonal cohort of HNSCC samples, providing consistency in the tissue analyzed for both the reference matrix as well as the orthogonal, deconvoluted dataset, and thereby avoiding errors from changes in expression profiles under different tumor microenvironments (even for the same cell type) [32,40,41].

Since these two deconvolution methods are based on different algorithms (support vector regression for CIBERSORTx and weighted non-negative least squares regression for MuSiC), we might observe minor discrepancies in the estimated cell proportions. In terms of the cell types that have a discrepancy between these two methods, we weighed the results from CIBERSORTx higher than that from MuSiC because of its improved performance in our benchmarking validation tests. Specifically, these two algorithms were both compared to the ground truth of our single-cell HNSCC dataset. CIBERSORTx showed a strong concordance between the deconvolution results and ground truth cell proportions [32], while MuSiC also indicated a good alignment between estimated and ground truth proportion (Figure 4 and Supplementary Figure S7). However, CIBERSORTx slightly outperformed MuSIC with median values of 0.97 and 0.98 for correlation co-efficiency in cell level and sample level validation tests respectively, compared to MuSiC, which showed best median values of 0.95 versus 0.97 under the same conditions. These evaluations suggest that CIBERSROTx outperforms MuSiC by a small margin, which guided us to use CIBERSORTx as the main exploratory tool and MuSiC as a secondary validation check. In addition, while MuSiC was unable to statistically separate CD8_conv_, CD8_exhaust_, and T_reg_ populations in terms of their effect on survival, CIBERSORTx analyses revealed a much more significant association of T_reg_ proportion with survival compared to these other T-cell subtypes. Thus, *in toto*, the combination of these two approaches emphasized a focus on T_reg_ for additional analyses.

The role of T_regs_ in HNSCC is somewhat controversial. Generally, T_regs_ are thought to suppress the anti-tumor immunity of T-cells and natural killer (NK) cells in some solid tumors and generally help to establish an immunosuppressive microenvironment [42,43,44]. Thus, T_reg_ infiltration is generally associated with a poor prognosis in many human carcinomas. For example, studies have reported that a high proportion of tumor-infiltrating T_regs_ was significantly associated with worse outcomes in breast cancer [45], hepatocellular carcinoma [46], lung cancer [42], gastric cancer [47], and ovarian cancer [48]. However, contradicting conclusions have been drawn recently concerning the prognostic value of T_regs_ in oncology, where it has been suggested that T_reg_ infiltration may have a positive effect on anti-tumor response. For example, high densities of tumor-infiltrating T_regs_ in colorectal carcinoma, malignant melanoma, and lymphoma are reported to be associated with improved outcomes [49,50,51,52]. In HNSCC, several studies have suggested that patients with high T_reg_ infiltration have significantly better overall survival [53,54,55,56,57]; however, these analyses utilized immunohistochemistry in a limited cohort to reach this conclusion. In addition, a few clinical correlative studies that used deconvolution methods in HNSCC have uncovered a favorable association between increased levels of T_reg_ infiltration and prognosis [11,58,59]. These studies, however, utilized older versions of CIBERSORT, and the signature matrix was based on sorted bulk data as opposed to single-cell sequencing data. Thus, there is precedent to support the findings of our study and to suggest that the effect of T_reg_ infiltration on tumor biology may be quite complex and specific to the disease context. Importantly, our findings offer a more rigorous approach to deconvolution, allowing for greater sample size than histology-based studies while offering improved precision over existing informatics approaches to date.

A possible explanation for the paradoxical observation of T_regs_ favorably affecting survival in HNSCC may relate to the potential translocation of microbial flora from the upper aerodigestive tract to HNSCC tissues [57], similar to theories proposed by Ladoire et al. for colorectal tumors [51]. It is possible that this microbiological hazard provokes a T-cell-mediated anti-microbial inflammatory response that involves Th17 cells and can thereby promote cancer growth. The Th17-cell-dependent pro-inflammatory and tumor-enhancing response can be attenuated by T_regs_, providing a possible explanation for the favorable role of T_regs_ in HNSCC prognosis. Certainly, future studies must rigorously examine the importance and relevance of T_regs_ and the oral microbiome using a well-controlled animal model to further develop and test these hypotheses. However, our data, which are validated using two orthogonal deconvolution approaches, seem to broadly belie the notion of cancer-specific effects of infiltrating immune cells.

We observed a strong clinical association between high expression of TNFRSF4 and improved survival. The TNF-receptor superfamily (TNFRSF) serves various key immunoregulatory functions and includes death receptors that trigger apoptosis in cancer cells and receptors that provide co-stimulatory signals to anti-tumor T-cells [60]. Targeting the TNFRSF is somewhat of a recent development among tumor immunotherapy approaches and shows promise for the treatment of cancer in preclinical studies when used in combination with chemotherapy or irradiation, which can induce immunogenic cell death and stimulate anti-tumor T-cell responses [61]. Indeed, various agonistic TNFRSF antibodies and recombinant forms of TNFSF ligands are under clinical evaluation [62,63,64]. With the success of immune checkpoint blockade therapies and promise of programmed death pathway-targeted agents, TNFRSF4 could represent a potential target for future therapeutics to control tumor progression in HNSCC.

Although the results from our deconvolution are useful, some limitations are noted. First, this study relies on scRNA-seq as well as bulk RNA-seq data acquired from representative biopsy specimens rather than entire tumors themselves. Thus, inherent heterogeneity of HNSCC tumors may prevent a single biopsy from adequately representing the full biological behavior of the tumor. However, it is noteworthy that despite this limitation, we appreciated highly significant associations between certain cell-type proportions and patient outcomes. Second, the deconvolution methods used herein do not take spatial distributions of cells into consideration. Thus, subtle localization differences that are more appreciable with immunohistochemistry approaches may be missed; yet, our approach also offers a statistical power that is not feasible to obtain with histology slides. Third, our approach provides relative proportions of cells rather than absolute cell counts, therefore not capturing the total lymphocytic infiltration in any given tumor. With advances in single-cell and genomic approaches [14], such as spatial transcriptomics, we expect these limitations of our analyses to be overcome and provide yet more robust data.

## 4. Methods

### 4.1. Bulk RNA-Seq Data and Clinical Information of HNSCC Tumors from TCGA

Bulk RNA-sequencing data of HNSCC tumors was obtained from The Cancer Genome Atlas (TCGA) database (https://portal.gdc.cancer.gov/ (accessed on 1 March 2020)). A total of 545 cases were selected using the following filtering criteria: Primary site = head and neck, disease type = squamous cell neoplasms, sample type = primary tumor, experimental strategy = RNA-seq, and workflow type = HTseq (High Throughput sequencing)counts or FPKM (Fragments Per Kilobase Million). The HTseq-FPKM normalized expression data was used as the input file for CIBERSORTx according to the published protocol [32], while HTseq-Counts expression data was used as the input file for MuSiC according to the previously described protocol [33]. Clinical information for HNSCC patients, including gender, diagnosis, age, clinical stage, T stage, lymph node involvement, pathological grade, smoking, survival status, and survival duration in months, were downloaded from cBioPortal for Cancer Genomics (http://www.cbioportal.org/ (accessed on 1 March 2020)). According to the publication guidelines, datasets may be used for publication without restriction or limitation, and accordingly, no IRB (Institutional Review Boards) approval was required for use of these de-identified data.

### 4.2. Single-Cell RNA-seq Data of HNSCC Tumors

scRNA-seq data was obtained from our previous publication [8]. We excluded one patient (MEEI5), which is one of the samples with matched primary and LN, in an effort to have a more uniform set of single-cell samples to generate signature matrices because the eventual pathology was determined to be a spindle cell carcinoma (SCC with spindle cell features). Briefly, we profiled transcriptomes of ∼5600 single cells by SMART-seq2 method [34] from 21 HNSCC samples, including 4 matched pairs of primary tumors and lymph node metastases [8]. Expression levels were quantified as Ei,j = log2(TPMi,j/10 + 1), where TPMi,j refers to transcript-per-million for gene i in sample j, as calculated by RSEM (RNA-Seq by Expectation-Maximization) [65]. Malignant cells were identified by a set of potential epithelial markers consisting of all cytokeratins, EPCAM (Epithelial Cellular Adhesion Molecule), and SFN (Stratifin), as well as the copy number variation (CNV) analysis. t-SNE analysis of the remaining non-malignant cells identified eight major clusters. Briefly, we defined the clusters by DBSCAN (Density-Based Spatial Clustering of Applications with Noise: parameters Epsilon = 3 and MinPoints = 5) using the normalized gene-count matrix. Clusters were assigned to cell types based on strong differentiation expression of known marker genes. The T-cell cluster was further subdivided into four subtypes, which were annotated based on the differential expression of T-cell markers to represent the main patterns of variability: CCR7 and TCF7 for conventional CD4 (CD4_conv_), GZMA/B/H/K and PRF1 for conventional CD8 (CD8_conv_), PD1, LAG3, TIGIT, and CTLA4 for exhausted CD8 (CD8_exhaust_), and FOXP3 and CD25 for T regulatory cells (T_regs_).

### 4.3. CIBERSORTx Deconvolution Analysis

We utilized the CIBERSORTx online tool implementation. We used the single-cell reference matrix file as previously described [8] to create a custom signature matrix. We applied two analysis modules to the bulk RNA-seq data, namely, cell fractions and gene expression. For cell fractions, we enumerated the proportions of distinct cell subpopulations in TCGA bulk tissue expression profiles. We imputed cell-type-specific expression profiles from bulk tissue transcriptomes using the High-Resolution mode for gene expression. This analysis provided estimates of sample-level gene expression variation among distinct cell types, which allowed exploring gene expression changes among distinct cellular subpopulations.

### 4.4. MuSiC Deconvolution Analysis

We inputted HTseq counts of the bulk RNA-seq data from TCGA as well as the multi-subject single-cell profiles from our scRNA-seq data in the MuSiC algorithm, implemented in R. Cell types from scRNA-seq were based on prior categorizations [8]. Genes in bulk data use Ensembl gene ID (Ensembl version 84) as their identifiers, whereas genes in the single-cell profiles use gene symbols. To be consistent with the bulk data, the Ensembl gene IDs of the genes in single-cell profiles were queried by using biomaRt package (version 2.42) [39,66]. The single-cell profiles were further filtered to keep the genes with a unique Ensembl gene ID. The filtered profiles then served as reference for estimating cell-type proportions of bulk data. By following the tutorial available on Github (https://xuranw.github.io/MuSiC/articles/MuSiC.html (accessed on 1 January 2020)), we obtained the estimated cell-type proportions for each sample by using the function music_prop. The estimated proportions were normalized to sum to 1 across included cell types.

### 4.5. Single-Cell RNA-seq Data of HNSCC Tumors

Patients were split at the median for each estimated cell type. The prognosis of each group of patients was examined by Kaplan–Meier survival, and log-rank tests compared the survival outcomes. Kaplan–Meier plots are presented for all the cell-type proportions, and the cell types with log-rank *p*-values less than 0.05 were defined as a prognostic cell type. Hazard ratios (HRs) and corresponding 95% confidence intervals (CIs) for risk of disease progression and mortality associated with high and low percentage of cell types were estimated using the Cox proportional-hazards model. Multivariable Cox model was adjusted for tumor stage, race, smoking status, and age. All statistical analyses were performed using R version 3.6.

## 5. Conclusions

In summary, we have shown through two distinct deconvolution methods of bulk RNA-seq data from >500 TCGA samples that higher proportions of tumor-infiltrating regulatory T-cells are associated with improved outcomes in HNSCC. Future studies may investigate the possibility of further deconvolution of regulatory T-cell subtypes as well as subtypes of other cells, such as dendritic cells, macrophages, and natural killer cells. Additionally, a more substantial understanding of the implications of regulatory T-cells in HNSCC may reveal unique prognostic approaches as well as potential therapeutic targets for more precise and effective treatments.

## Figures and Tables

**Table 1:** Cox proportional-hazard regression analysis for survival and T-cell proportions estimated by CIBERSORTx and MuSiC (prop., proportion; HR, hazard ratio; CI, confidence interval; ref, reference).

Variables	CIBERSORTx			MuSiC		
	HR	95% CI	*p*	HR	95% CI	*p*
**Cell type prop.**						
T-cell low (ref)	1.00			1.00		
T-cell high	0.63	0.47–0.83	0.001	0.71	0.53–0.93	0.014
**Stage**						
Stage I (ref)	1.00			1.00		
Stage II	1.50	0.58–3.90	0.404	1.49	0.57–3.87	0.415
Stage III	1.83	0.71–4.71	0.21	1.80	0.70–4.63	0.224
Stage IVA	2.53	1.03–6.21	0.043	2.45	1.00–6.04	0.051
Stage IVB	5.53	1.66–18.39	0.005	5.56	1.67–18.51	0.005
Stage IVC	17.61	1.92–161.32	0.011	18.38	2.00–168.47	0.01
Not reported	2.21	0.84–5.81	0.107	2.05	0.78–5.38	0.145
**Race**						
White (ref)	1.00			1.00		
Black	1.39	0.86–2.26	0.18	1.36	0.84–2.21	0.212
Hispanic	1.53	0.85–2.75	0.159	1.46	0.81–2.63	0.21
Other	1.11	0.56–2.19	0.761	1.18	0.60–2.33	0.631
**Smoke**						
Never (ref)	1.00			1.00		
Ever	0.88	0.63–1.22	0.437	0.88	0.63–1.22	0.446
**Age**	1.02	1.00–1.03	0.007	1.02	1.01–1.03	0.004

**Table 2:** Cox proportional-hazard regression analysis for survival and B-cell proportions estimated by CIBERSORTx and MuSiC (prop., proportion; HR, hazard ratio; CI, confidence interval; ref, reference).

Variables	CIBERSORTx			MuSiC		
	HR	95% CI	*p*	HR	95% CI	*p*
**Cell type prop.**						
B-cell low (ref)	1.00			1.00		
B-cell high	0.59	0.45–0.79	3 × 10^−4^	0.69	0.44–1.11	0.126
**Stage**						
Stage I (ref)	1.00			1.00		
Stage II	1.57	0.60–4.08	0.353	1.67	0.64–4.34	0.293
Stage III	1.93	0.75–4.96	0.173	1.94	0.76–4.99	0.168
Stage IVA	2.71	1.11–6.65	0.029	2.72	1.11–6.66	0.029
Stage IVB	5.95	1.79–19.74	0.004	6.75	2.03–22.44	0.002
Stage IVC	27.77	3.03–254.86	0.003	21.37	2.34–194.94	0.007
Not reported	2.34	0.89–6.14	0.085	2.18	0.83–5.73	0.114
**Race**						
White (ref)	1.00			1.00		
Black	1.57	0.97–2.55	0.069	1.46	0.9–2.37	0.127
Hispanic	1.54	0.86–2.78	0.15	1.52	0.84–2.74	0.163
Other	1.08	0.55–2.12	0.834	1.09	0.56–2.16	0.795
**Smoke**						
Never (ref)	1.00			1.00		
Ever	0.85	0.61–1.19	0.343	0.88	0.63–1.22	0.432
**Age**	1.02	1.01–1.03	0.003	1.02	1.01–1.03	0.006

**Table 3:** Cox proportional-hazard regression analysis for survival and T_reg_ proportions estimated by CIBERSORTx and MuSiC (prop., proportion; HR, hazard ratio; CI, confidence interval; ref, reference).

Variables	CIBERSORTx			MuSiC		
	HR	95% CI	*p*	HR	95% CI	*p*
**Cell type prop.**						
T_reg_ low (ref)	1.00			1.00		
T_reg_ high	0.61	0.46–0.80	4 × 10^−4^	0.70	0.52–0.95	0.021
**Stage**						
Stage I (ref)	1.00			1.00		
Stage II	1.75	0.67–4.54	0.252	1.54	0.59–4.01	0.372
Stage III	2.05	0.79–5.26	0.138	1.86	0.72–4.79	0.196
Stage IVA	2.87	1.17–7.05	0.022	2.59	1.06–6.36	0.038
Stage IVB	6.21	1.87–20.63	0.003	5.87	1.77–19.46	0.004
Stage IVC	31.03	3.36–286.33	0.002	18.37	2.01–168.11	0.01
Not reported	2.51	0.95–6.61	0.064	2.05	0.78–5.37	0.146
**Race**						
White (ref)	1.00			1.00		
Black	1.46	0.90–2.37	0.126	1.52	0.94–2.48	0.089
Hispanic	1.48	0.82–2.67	0.191	1.55	0.86–2.79	0.148
Other	1.06	0.54–2.10	0.862	1.09	0.56–2.15	0.796
**Smoke**						
Never (ref)	1.00			1.00		
Ever	0.87	0.62–1.21	0.409	0.85	0.61–1.18	0.335
**Age**	1.02	1.01–1.03	0.006	1.02	1.01–1.03	0.005

**Table 4:** Cox proportional-hazard regression analysis for survival and TNFRSF4 expression estimated by bulk RNA-seq and CIBERSORTx (prop., proportion; HR, hazard ratio; CI, confidence interval; ref, reference).

Variables	Bulk RNAseq			CIBERSORTx—T_reg_		
	HR	95% CI	*p*	HR	95% CI	*p*
**Cell type prop.**						
TNFRSF4 low (ref)	1.00			1.00		
TNFRSF4 high	0.57	0.43–0.75	8 × 10^−5^	0.59	0.46–0.75	2 × 10^−5^
**Stage**						
Stage I (ref)	1.00			1.00		
Stage II	2.31	0.88–6.02	0.088	2.03	0.88–4.70	0.097
Stage III	1.54	0.59–4.00	0.376	1.60	0.72–3.56	0.249
Stage IVA	1.78	0.69–4.59	0.232	1.87	0.84–4.17	0.128
Stage IVB	2.65	1.08–6.52	0.033	2.64	1.23–5.65	0.013
Stage IVC	4.79	1.44–15.92	0.011	5.28	1.76–15.84	0.003
Not reported	16.43	1.80–150.11	0.013	22.36	2.59–193.19	0.005
**Race**						
White (ref)	1.00			1.00		
Black	1.55	0.96–2.49	0.073	1.55	1.00–2.41	0.05
Hispanic	1.55	0.88–2.74	0.128	1.64	0.98–2.73	0.058
Other	1.10	0.55–2.17	0.792	1.21	0.69–2.13	0.51
**Smoke**						
Never (ref)	1.00			1.00		
Ever	0.83	0.59–1.15	0.258	0.74	0.55–0.98	0.036
**Age**	1.02	1.00–1.03	0.009	1.02	1.01–1.03	0.002

## Data Availability

Raw expression data is available through The Cancer Genome Atlas (TCGA) database (https://portal.gdc.cancer.gov/ (accessed on 1 March 2020)). Processed data is available through Supplementary files. Analysis scripts are available upon request from the corresponding author.

## Supplementary Materials

The following are available online at https://www.mdpi.com/2072-6694/13/6/1230/s1, Figure S1: (A) Heatmap of the signature matrix of the 9 major cell types by CIBERSORTx. (B) Heatmap of the signature matrix of 12 cell types (8 major cell types and 4 T-cell subtypes) by CIBERSORTx. Figure S2: Heatmaps of immune cell-type proportions estimated by CIBERSORTx. The cell-type proportions are ordered from high (top) to low (bottom). The tumor stage and tissue origin are annotated as side bars. The immune cells are (A) T cell, (B) B cell, (C) Dendritic, and (D) Macrophage. Figure S3: Heatmaps of non-immune cell-type proportions estimated by CIBERSORTx. The cell-type proportions are ordered from high (top) to low (bottom). The tumor stage and tissue origin are annotated as side bars. The cell types are (A) Malignant cell, (B) Fibroblast, (C) Endothelial, (D) Myocyte, and (E) Mast. Figure S4: Heatmaps of immune cell-type proportions estimated by CIBERSORTx. The cell-type proportions are ordered from high (top) to low (bottom). The tumor subtype is annotated as a side bar. The immune cells are (A) T cell, (B) B cell, (C) Dendritic, and (D) Macrophage. Figure S5: Heatmaps of non-immune cell-type proportions estimated by CIBERSORTx. The cell-type proportions are ordered from high (top) to low (bottom). The tumor subtype is annotated as a side bar. The cell types are (A) Malignant cell, (B) Fibroblast, (C) Endothelial, (D) Myocyte, and (E) Mast. Figure S6: Correlation plot of estimated T cell and B cell proportions by CIBERSORTx. Both x- and y-axes are in log2 scale. Figure S7: (A) Comparison of ground-truth cell proportions with the estimated proportions by MuSiC for all HNSCC samples. The validation run used a combination of 14 (reference) and 7 (validation). The ground-truth and estimated cell proportions are paired for each sample and demarcated by black and red lines, respectively. The sample tumor numbers shown with an asterisk indicate a metastatic sample. (B) Concordance between cell-type proportions measured by scRNA-seq (ground truth) and MuSiC for all HNSCC samples. The validation run used a combination of 14 (reference) and 7 (validation). The correlation is calculated by Pearson method and samples have the same naming criteria as in (A). (C) and (D) Bar plot of the Pearson correlation coefficient (r) between cell-type proportions measured by scRNA-seq and MuSic deconvolution for nine cell types. The validation run uses a combination of either 14 (reference) and 7 (validation) (C) or 18 (reference) and 3 (validation) (D). Figure S8: (A) Heatmap of the relative cell fractions of the 9 major cell types for each patient estimated by MuSiC. (B) Association between cell proportions and overall survival in patients with HNSCC profiled by TCGA. Estimated cell proportions were stratified by a half–half split, and the separation between survival curves was evaluated using a log-rank test. Figure S9: (A) Histogram of Pearson correlation coefficient (r) between cell-type proportions estimated by CIBERSORTx and MuSiC for all samples in the study. (B) Bar plot of the Pearson correlation coefficient (r) between cell-type proportions estimated by CIBERSORTx and MuSiC for nine cell types. Figure S10: Association between gene expression and overall survival in patients with HNSCC profiled by TCGA. The gene expression is measured by bulk-RNA-seq and was stratified by a half–half split. The separation between survival curves was evaluated using a log-rank test. File S1: Cell proportions of the nine major cell types estimated by CIBERSORTx. File S2: Cell proportions of the 12 cell types (eight major cell types and four T-cell subtypes) estimated by CIBERSORTx. File S3: Cell proportions of the nine major cell types estimated by MuSiC. File S4: Cell proportions of the 12 cell types (eight major cell types and four T-cell subtypes) estimated by MuSiC. File S5: Imputed gene expression for 33 marker genes by CIBERSORTx high-resolution mode.
