# Supplementary material for: Single-Cell Deconvolution of Head and Neck Squamous Cell Carcinoma
Source: Cancers (Basel). 2021 Mar 11;13(6):1230. doi: 10.3390/cancers13061230 (PMC7999850; doi:10.3390/cancers13061230)
Supplement: Supplementary file 1 [file cancers-13-01230-s001.zip › for_cancers/0_deconvolution_supplement_v4_cancers.docx]

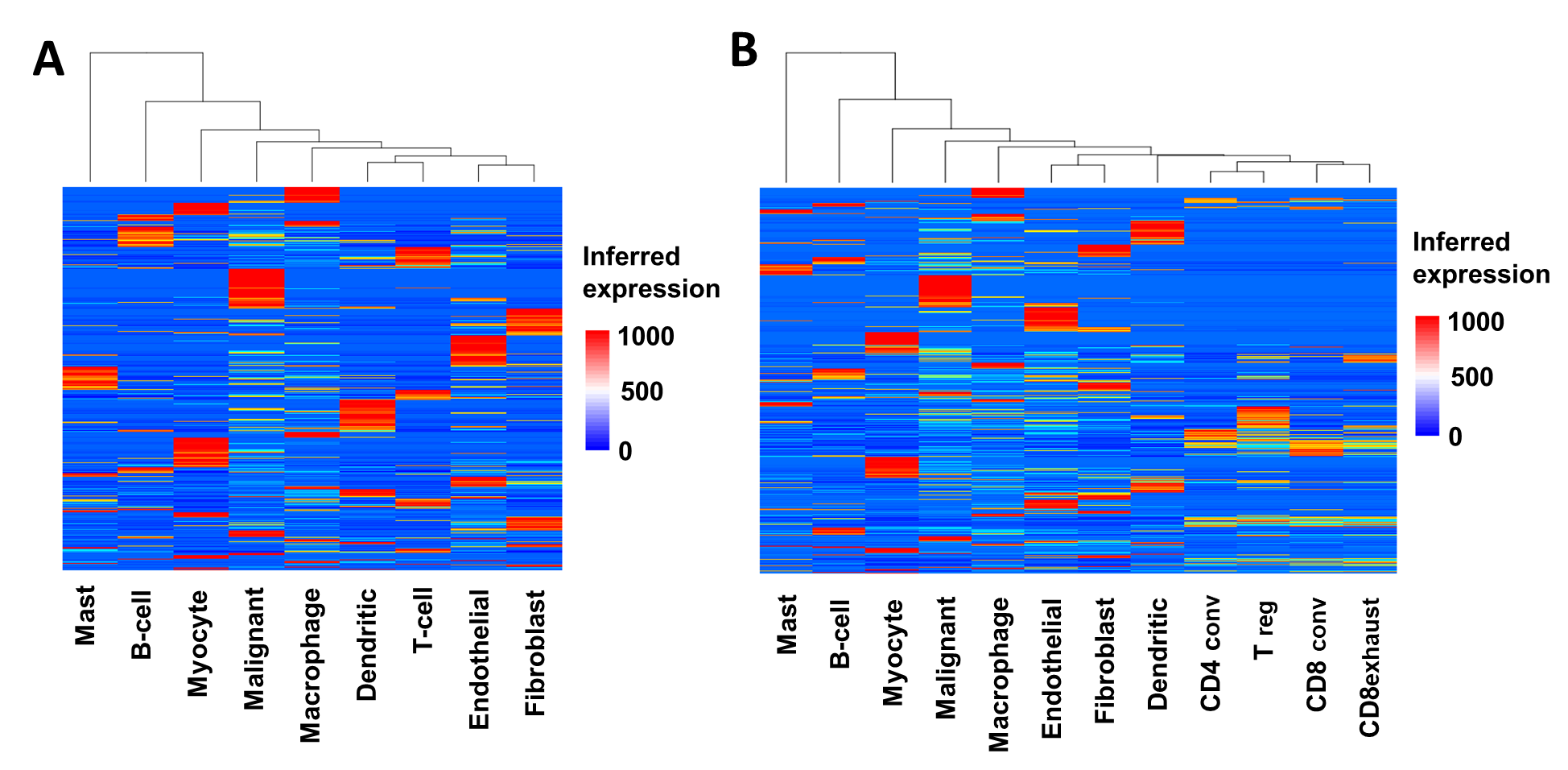


**Figure S1. (A)** Heatmap of the signature matrix of the 9 major cell types by CIBERSORTx. **(B)** Heatmap of the signature matrix of 12 cell types (8 major cell types and 4 T-cell subtypes) by CIBERSORTx.


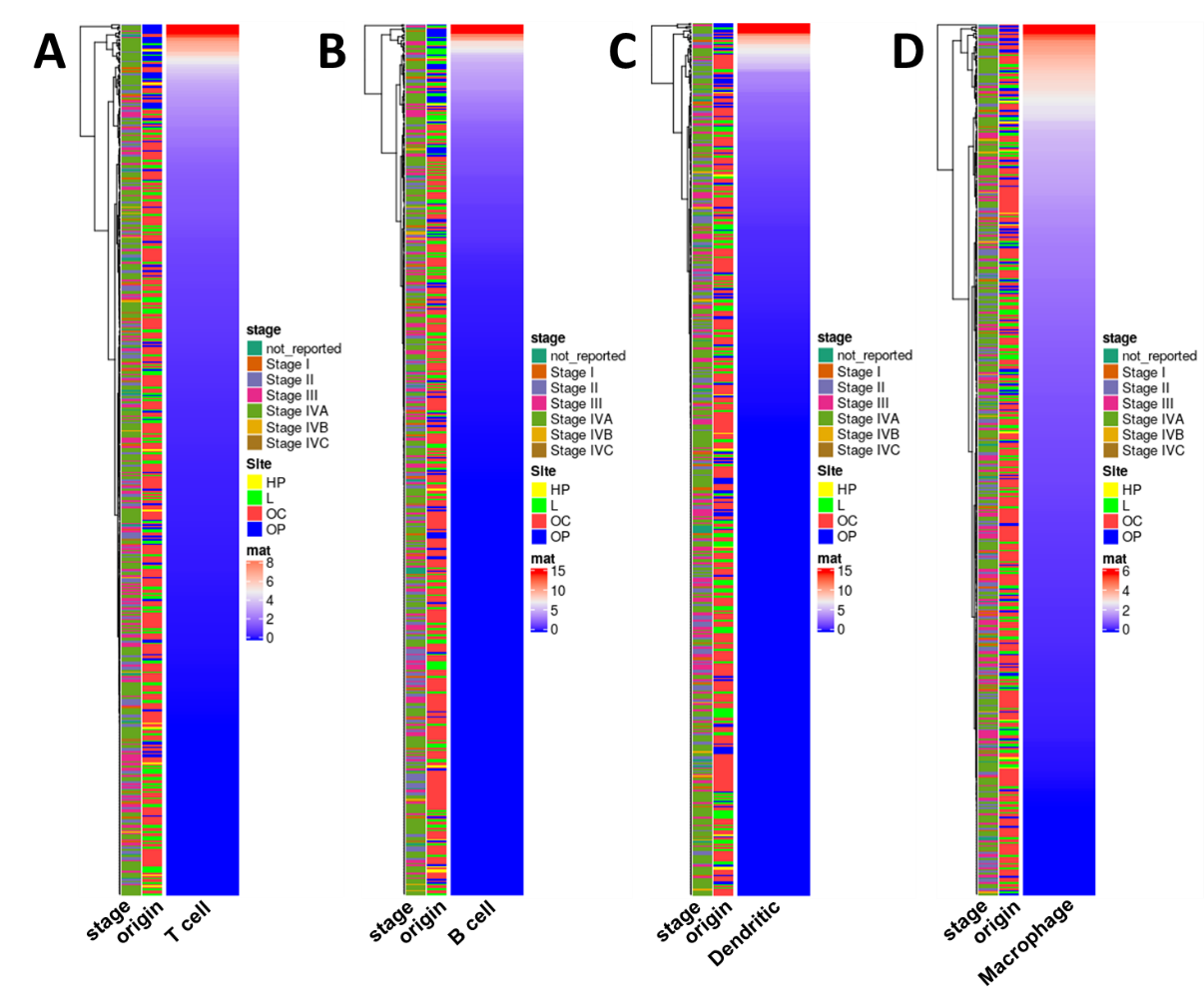


**Figure S2**. Heatmaps of immune cell type proportions estimated by CIBERSORTx. The cell type proportions are ordered from high (top) to low (bottom). The tumor stage and tissue origin are annotated as side bars (HP=Hypopharynx; L=Larynx; OC=Oral Cavity; OP=Oropharynx). The immune cells are **(A)** T cell, **(B)** B cell, **(C)** Dendritic, and **(D)** Macrophage.


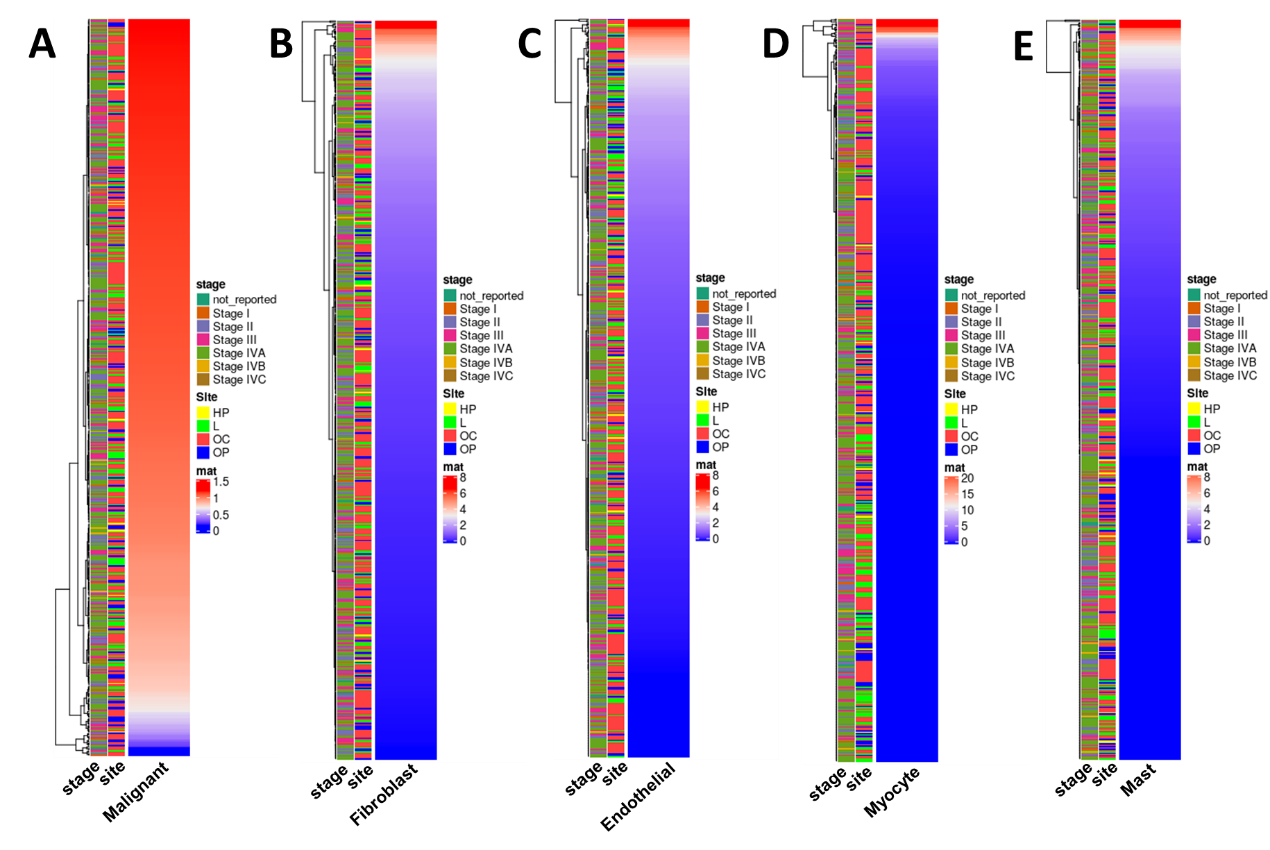


**Figure S3**. Heatmaps of non-immune cell type proportions estimated by CIBERSORTx. The cell type proportions are ordered from high (top) to low (bottom). The tumor stage and tissue origin are annotated as side bars (HP=Hypopharynx; L=Larynx; OC=Oral Cavity; OP=Oropharynx). The cell types are **(A)** Malignant cell, **(B)** Fibroblast, **(C)** Endothelial, **(D)** Myocyte, and **(E)** Mast.


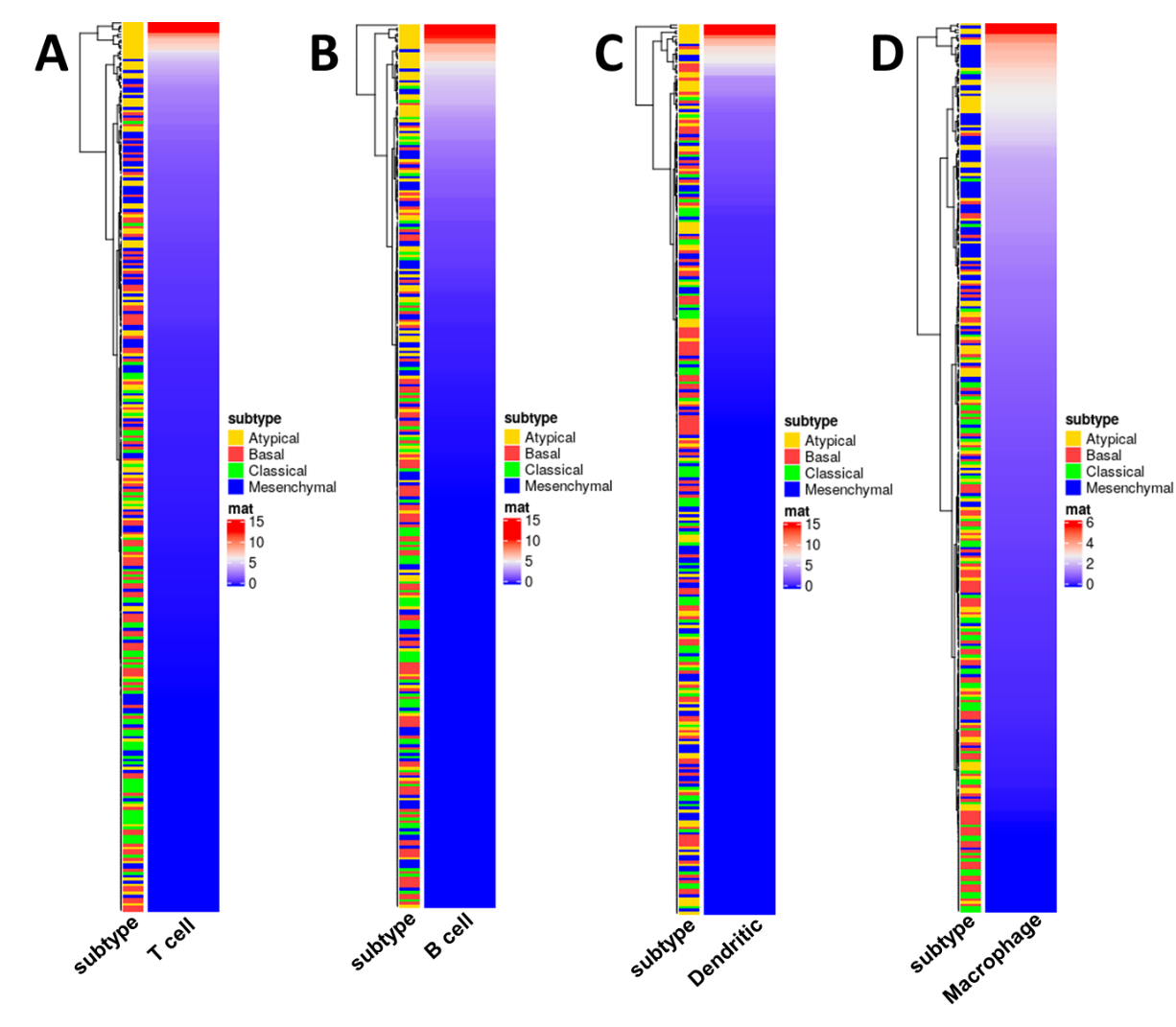


**Figure S4**. Heatmaps of immune cell type proportions estimated by CIBERSORTx. The cell type proportions are ordered from high (top) to low (bottom). The tumor subtype is annotated as a side bar. The immune cells are **(A)** T cell, **(B)** B cell, **(C)** Dendritic, and **(D)** Macrophage.


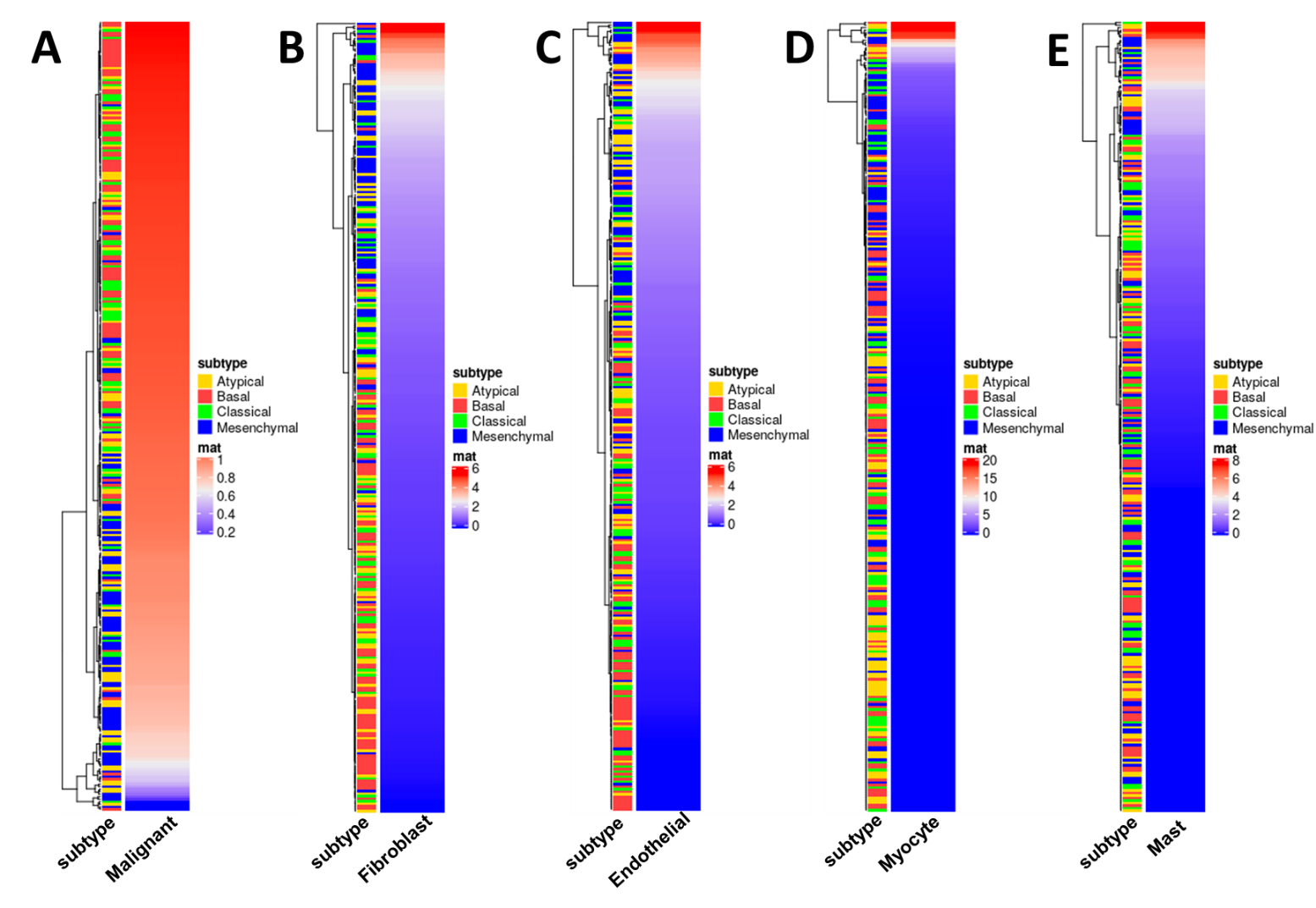


**Figure S5**. Heatmaps of non-immune cell type proportions estimated by CIBERSORTx. The cell type proportions are ordered from high (top) to low (bottom). The tumor subtype is annotated as a side bar. The cell types are **(A)** Malignant cell, **(B)** Fibroblast, **(C)** Endothelial, **(D)** Myocyte, and **(E)** Mast.


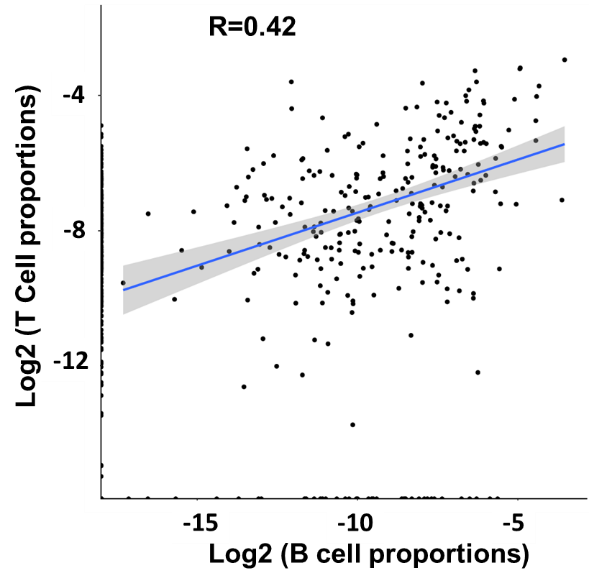


**Figure S6**. Correlation plot of estimated T cell and B cell proportions by CIBERSORTx. Both x- and y-axes are in log2 scale.

**
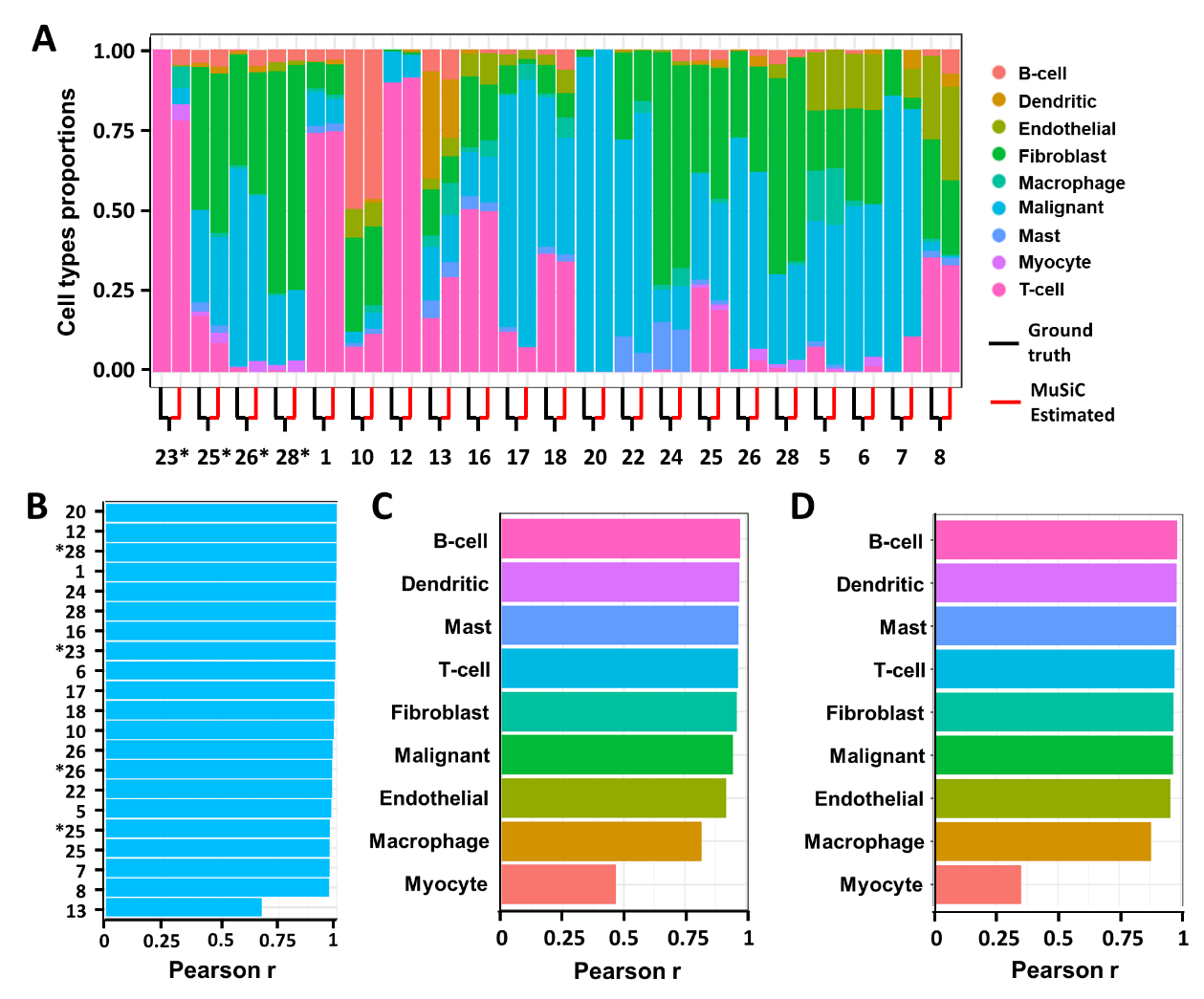
**

**Figure S7. (A)** Comparison of ground-truth cell proportions with the estimated proportions by MuSiC for all HNSCC samples. The validation run used a combination of 14 (reference) and 7 (validation). The ground-truth and estimated cell proportions are paired for each sample and demarcated by black and red lines, respectively. The sample tumor numbers shown with an asterisk indicate a metastatic sample. **(B)** Concordance between cell type proportions measured by scRNA-seq (ground truth) and MuSiC for all HNSCC samples. The validation run used a combination of 14 (reference) and 7 (validation). The correlation is calculated by Pearson method and samples have the same naming criteria as in **(A)**. **(C)** and **(D)** Bar plot of the Pearson correlation coefficient (r) between cell type proportions measured by scRNA-seq and MuSic deconvolution for nine cell types. The validation run uses a combination of either 14 (reference) and 7 (validation) **(C)** or 18 (reference) and 3 (validation) **(D)**.

**
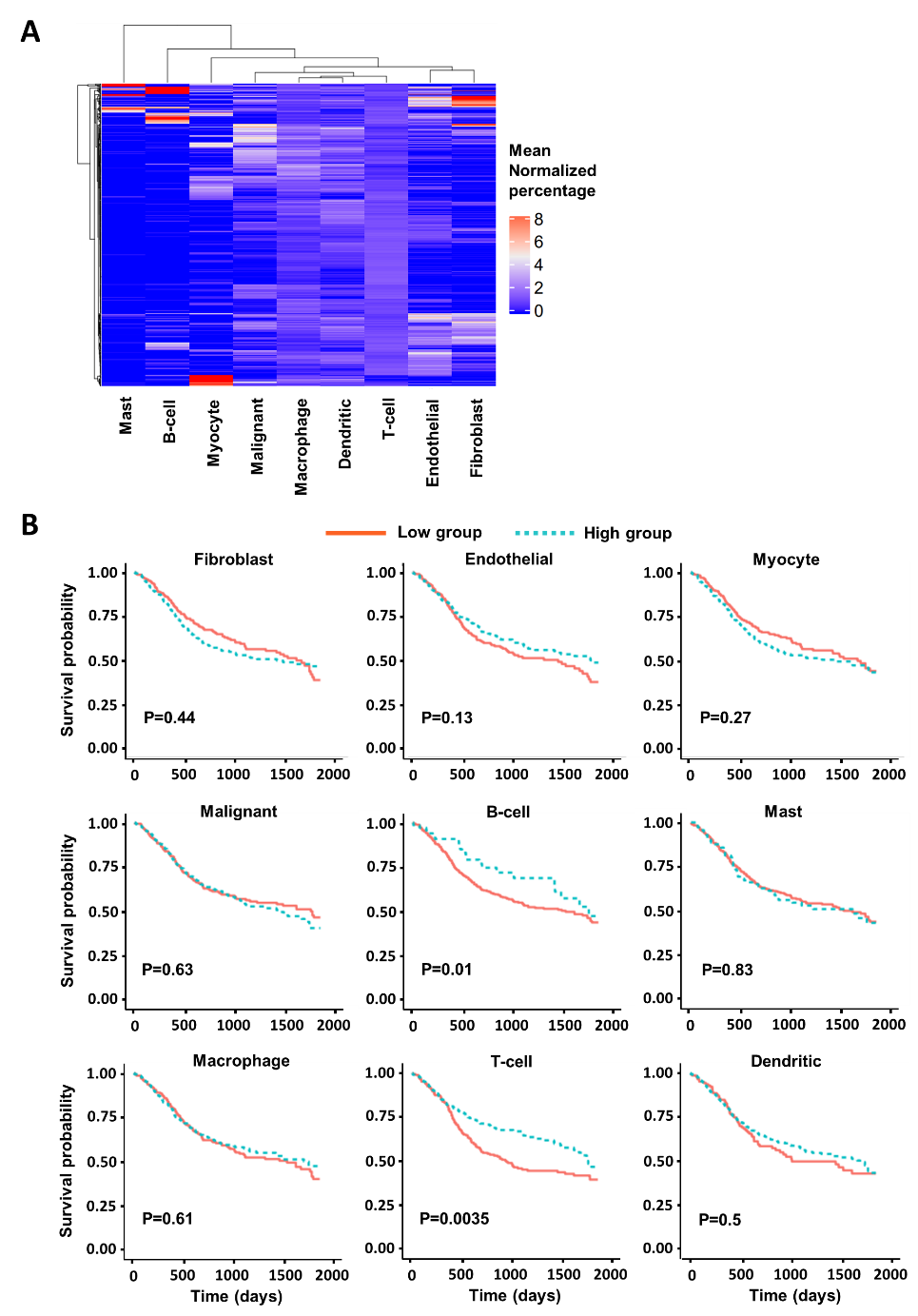
**

**Figure S8. (A)** Heatmap of the relative cell fractions of the 9 major cell types for each patient estimated by MuSiC. **(B)** Association between cell proportions and overall survival in patients with HNSCC profiled by TCGA. Estimated cell proportions were stratified by a half-half split, and the separation between survival curves was evaluated using a log-rank test.

**
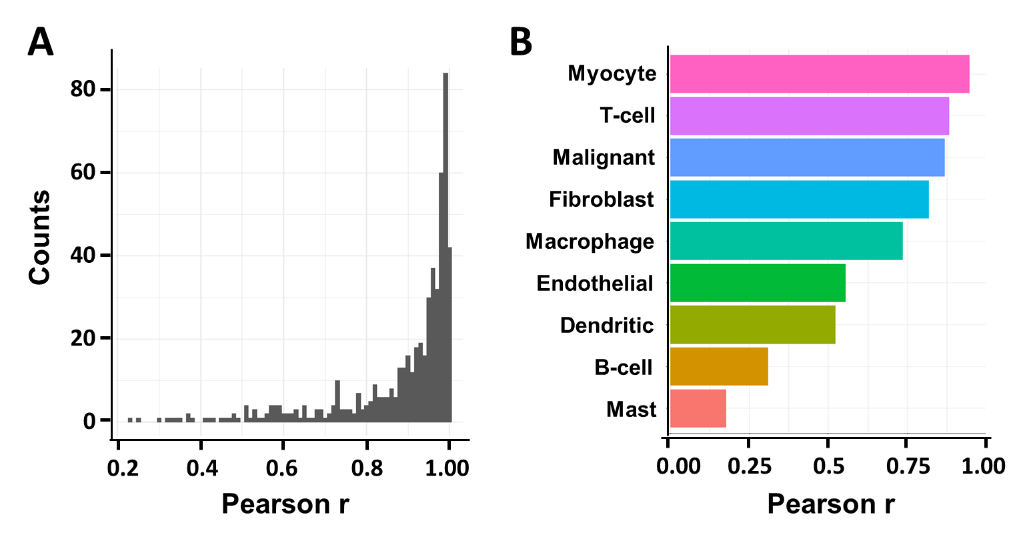
**

**Figure S9. (A)** Histogram of Pearson correlation coefficient (r) between cell type proportions estimated by CIBERSORTx and MuSiC for all samples in the study. **(B)** Bar plot of the Pearson correlation coefficient (r) between cell type proportions estimated by CIBERSORTx and MuSiC for nine cell types.

**
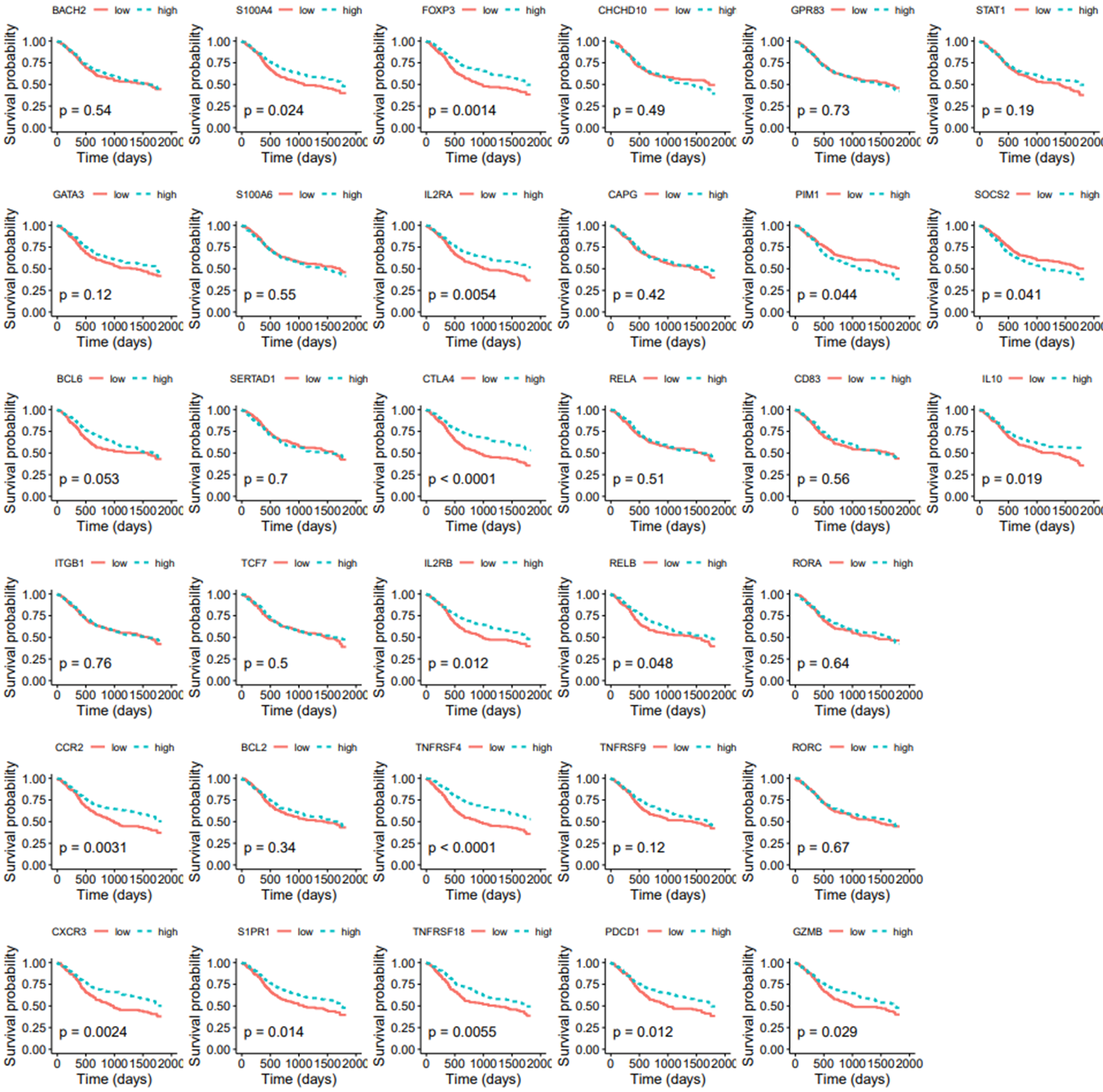
**

**Figure S10.** Association between gene expression and overall survival in patients with HNSCC profiled by TCGA. The gene expression is measured by bulk-RNA seq and was stratified by a half-half split. The separation between survival curves was evaluated using a log-rank test.

**File S1. Cell proportions of the nine major cell types estimated by CIBERSORTx.**

**File S2. Cell proportions of the 12 cell types (eight major cell types and four T-cell subtypes) estimated by CIBERSORTx.**

**File S3. Cell proportions of the nine major cell types estimated by MuSiC.**

**File S4. Cell proportions of the 12 cell types (eight major cell types and four T-cell subtypes) estimated by MuSiC.**

**File S5. Imputed gene expression for 33 marker genes by CIBERSORTx high-resolution mode**
